# Factors influencing the knowledge, attitude, and practices of police personnel toward dengue fever in Kathmandu, Nepal

**DOI:** 10.1002/1348-9585.12421

**Published:** 2023-09-04

**Authors:** Damodar Paudel, Sampurna Kakchapati, Nabin Lageju, Samriddhi Karki, Jayanti Dhungana, Sirish Regmi, Deepa Chudal, Ram Prasad Sharma

**Affiliations:** ^1^ Department of Medicine Nepal Police Hospital Kathmandu Nepal; ^2^ HERD International Kathmandu Nepal

**Keywords:** attitude, dengue, knowledge, Nepal, police personnel, practices

## Abstract

**Objective:**

Dengue fever is a significant public health problem in Nepal, and police personnel are considered to play a crucial role in preventing and controlling dengue fever. This study aimed to assess the factors that influence the knowledge, attitudes, and practices of police personnel toward dengue in Kathmandu, Nepal.

**Methods:**

The study design was a descriptive cross‐sectional study among 422 police personnel, where data were collected using self‐administered questionnaires. Bi‐variate analysis and multivariate analysis were used to examine the association between sociodemographic factors and environmental factors with knowledge, attitude, and practices of dengue.

**Results:**

The study found that the knowledge, attitude, and practice toward dengue prevention was 58%, 46%, and 75%, respectively. The study found that family history of dengue (AOR = 2.78, 95% CI = 1.38‐5.6), owning bed nets (AOR = 2.13, 95% CI = 1.04‐4.35) and having covered water storage containers (AOR = 2.99, 95% CI = 1.74‐5.13) were associated with higher odds of knowledge on dengue. Having family history of dengue (AOR = 2.45, 95% CI = 1.24‐4.87) and the presence of broken glasses or discarded plastic bottles in the house (AOR = 2.07, 95% CI = 1.93‐5.36) were associated with attitude on dengue. Knowledge on dengue was associated with higher odds of attitude (AOR = 3.3, 95% CI = 2.09‐5.36) and practices (AOR = 3.21, 95% CI = 1.93, 5.36).

**Conclusion:**

The study identified specific factors associated with knowledge, attitude, and practices toward dengue prevention. The study concluded that regular training and awareness‐raising activities are needed to improve their knowledge, attitudes, and practices toward dengue.

## 1 INTRODUCTION

Dengue is a health problem globally. It is an Arboviral disease and one of the most common public health threats, neglected viral disease in the tropics and subtropics. People once infected with the dengue virus can get immunity lifelong but there is no cross protective immunity. It is worldwide distributed in the tropics, 390 million people infected with dengue every year and only 96 million people manifest with the symptoms.^1^ *Aedes aegypti* and *Aedes albopictus* mosquitoes are the main vectors of the disease. The distribution of *A. aegypti* is predominantly increasing in the tropics because of the urbanization and the increment of the biodegradable product like as plastic and the tier, where larva of the mosquito can find the habitat.^2^, ^3^ Person can get dengue virus four times in his lifetime. Changing environment and global warming, huge increasing mosquitoes and case number are challenging to face the epidemic for the upcoming years.^4^

Kathmandu Valley has registered a huge spurt in dengue cases, 12 654 people infected with dengue as of September 4, 2002.^5^ Hospitals in the Kathmandu Valley have reported a massive surge in dengue cases. Knowledge toward emerging infectious organisms and mechanism of transmission is essential to prevent from spreading of dengue diseases.^6^, ^7^ Lack of knowledge and practice about dengue lead to high risk of disease spreading causes increasing morbidity among police personnel of Kathmandu Valley. Also, the police barracks were favor for the dengue transmission and mosquito proliferation. Moreover, Ministry of Health and Population to seek the help of police to combat dengue outbreak spread in 42 districts of the country that may risk to be infected with dengue.

Lack of effective mosquito control; demographic changes including uncontrolled urbanization often accompanied by substandard housing, poor water supplies, and inadequate sewage and waste management; increase in travel and commerce; and poor public health infrastructure are risk for dengue fever.^8^, ^9^ In Nepal, dengue is a rapidly emerging disease. The number of cases increased rapidly with the data of mortality in all provinces at the end of the rainy season.^10^, ^11^ Loss of duty days and erosion of the mission capability in combatants are underestimated. Due to their job nature and station in the barracks, they are more vulnerable to get infected with the disease.

Thus, in order to improve and design sustainable public health interventions for dengue throughout the police personnel of Nepal, with people having different socioeconomic and cultural backgrounds, it is essential to recognize and understand the people's knowledge, attitude, and practices (KAP) on dengue virus and its vectors.^7^, ^12^, ^13^ While several KAP studies have been conducted in Nepal, these were limited to specific dengue‐endemic areas or only focused on dengue‐infected people.^11^, ^12^, ^13^ Furthermore, none of the previous studies were conducted among police personnel that may be risk of dengue. To our knowledge, there is no previous population based study done among police personnel to assess knowledge, attitude, and practice to dengue fever, as this is an important effort in designing anti dengue intervention programs. And also, the police personnel have a gap of knowledge and understanding of the causes, transmission, and preventive practice of the dengue which impacts on health and combat readiness among police personnel. Therefore, this study was focused on knowledge, attitude, and practice and its associated factors toward dengue fever and the study helped to effective preventive intervention of police personnel related to dengue. The study is useful for the police personnel to evaluate and plan to increase knowledge and perception related to dengue fever in terms of prevention and control of the disease. Assist the police personnel in the study areas to develop appropriate health education methods to improve army personnel's knowledge.

## 2 METHODS

A descriptive cross‐sectional study was conducted to identify the knowledge, attitude, and practice regarding dengue fever among police personnel of Kathmandu valley. With standard formula, the police personnel size of 411 police personnel estimated; given that the margin of error alpha (*α*) = 0.05, prevalence = 0.05, the confidence level is = 95%, design effect = 1.5, and the non‐response rate = 5%. There were 10 police barracks in the Kathmandu valley and among them, 411 police personnel were selected by systematic random sampling. Self‐administrated questionnaire was used for data collection adapted from different literatures^11^, ^12^, ^13^ and consultation with experts. Data collection tool consisted of five parts. Part I dealt with sociodemographic information, Part II dealt with environment factors, Part III dealt with information on dengue knowledge including causative agent, transmission, Part IV dealt with attitude on dengue, and Part V dealt with practice on dengue prevention. Knowledge, attitude, and practice score was obtained by summing up the responses of each item of subscale to derive the composite value out of a possible total score of 20 for knowledge, seven for attitude, and none for practice. Knowledge was defined as “adequate” or “inadequate,” attitude and practice were defined as “good” or “poor” based on a 70% cut‐off point. Bi‐variate analysis (Chi‐squared test and Independent *t*‐test) was used to examine the association between sociodemographic factors, environment factors with knowledge, attitude and practice regarding dengue among police personnel. A *P*‐value less than .05 was considered as statistically significant. Initially, explanatory determinants were included in the model one at a time to examine their univariate relationship with the outcome and variables that were significant in the univariate analysis at *P*‐value <.20 were fitted in the multivariate analysis. Multivariate analysis with all independent variables entered at the same time was completed to adjust for the effect of confounding and adjusted OR and 95% CI were computed. Multicollinearity was checked among the variables, and there was no significant collinearity (variance inflation factor 1–2). The Hosmer‐Lemeshow test was performed to test the goodness‐of‐fit of the multivariate logistic regression model, and the model was found to be a good fit (*P* > .05). All statistical analyses and graphs for this study were conducted using the R program. Ethical clearance was obtained from Nepal Police Hospital, Institutional Review Committee of Nepal Health Research Council for this study with registration number IRC 06/2079.

## 3 RESULTS

Table 1 shows the sociodemographic characteristics and environmental related factors among police personnel. The majority of the police personnel were males (88.3%), followed by females (11.7%), and the mean age was 31.8 years (*SD* = 9.7 years). The majority of the police personnel were Hindu (93.4%), and the remaining police personnel belonged to Buddhist (3.9%) and other (2.7%) religions. In terms of caste, the largest group was Janajatis (38.7%), followed by Dalits (25.8%), Upper Caste Groups (15.8%), Madhesi (9.5%), and Others (10.2%). The mean number of family members was 5.9 (*SD* = 2.3). In terms of education, the majority of the police personnel had proficiency level certificate and above (57.4%), while the remaining police personnel had SEE/SLC and below (42.6%). The majority of the police personnel owned a bed net (91.2%), and most of them did not have a history of dengue among family members (87. 6%). Regarding household‐related factors, most of the police personnel had a good drainage system inside their household (73%), natural light inside the house (88.8%), and regular water supply (90.5%). However, many police personnel had stored water in containers (37.5%), and some of these containers were not covered tightly (26.8%). The majority of the police personnel had flower vases in their house (66.4%), while a smaller proportion had discarded tires around the house (18%) or broken glasses/discarded plastic bottles inside the house (23.1%). Similarly, the majority of the police personnel lacked garbage disposal nearby their house (62.5%), while the remaining police personnel had such facilities nearby.

**TABLE 1:** Sociodemographic characteristics and environment related characteristics of police personnel.

Characteristics	Number (*N* = 411)	Percent
Gender		
Male	363	88.3
Female	48	11.7
Age		
Mean (*SD*)	31.8 (9.7)	
Religion		
Hindu	384	93.4
Buddhist	16	3.9
Others	11	2.7
Caste		
Upper caste groups	65	15.8
Janajatis	159	38.7
Dalits	106	25.8
Madhesi	39	9.5
Others	42	10.2
Number of family member		
Mean (*SD*)	5.9 (2.3)	
Education		
SEE/SLC and below	175	42.6
Proficiency level certificate and above	236	57.4
Ownership of bed net		
No	36	8.8
Yes	375	91.2
History of dengue among family members		
No	360	87.6
Yes	51	12.4
Good drainage system inside household		
No	111	27
Yes	300	73
Natural light inside the house		
No	46	11.2
Yes	365	88.8
The house has stored water in containers		
No	257	62.5
Yes	154	37.5
The water storage containers were covered tight		
No	110	26.8
Yes	301	73.2
Presence of flower vases in the house		
No	138	33.6
Yes	273	66.4
Presence of discarded tires around the house		
No	337	82
Yes	74	18
Broken glasses/discarded plastic bottles were present in the house		
No	316	76.9
Yes	95	23.1
Regular water supply		
No	39	9.5
Yes	372	90.5
Availability of public water tap nearby		
No	203	49.4
Yes	208	50.6
Presence of garbage disposal nearby the house		
No	257	62.5
Yes	154	37.5

**FIGURE 1:**
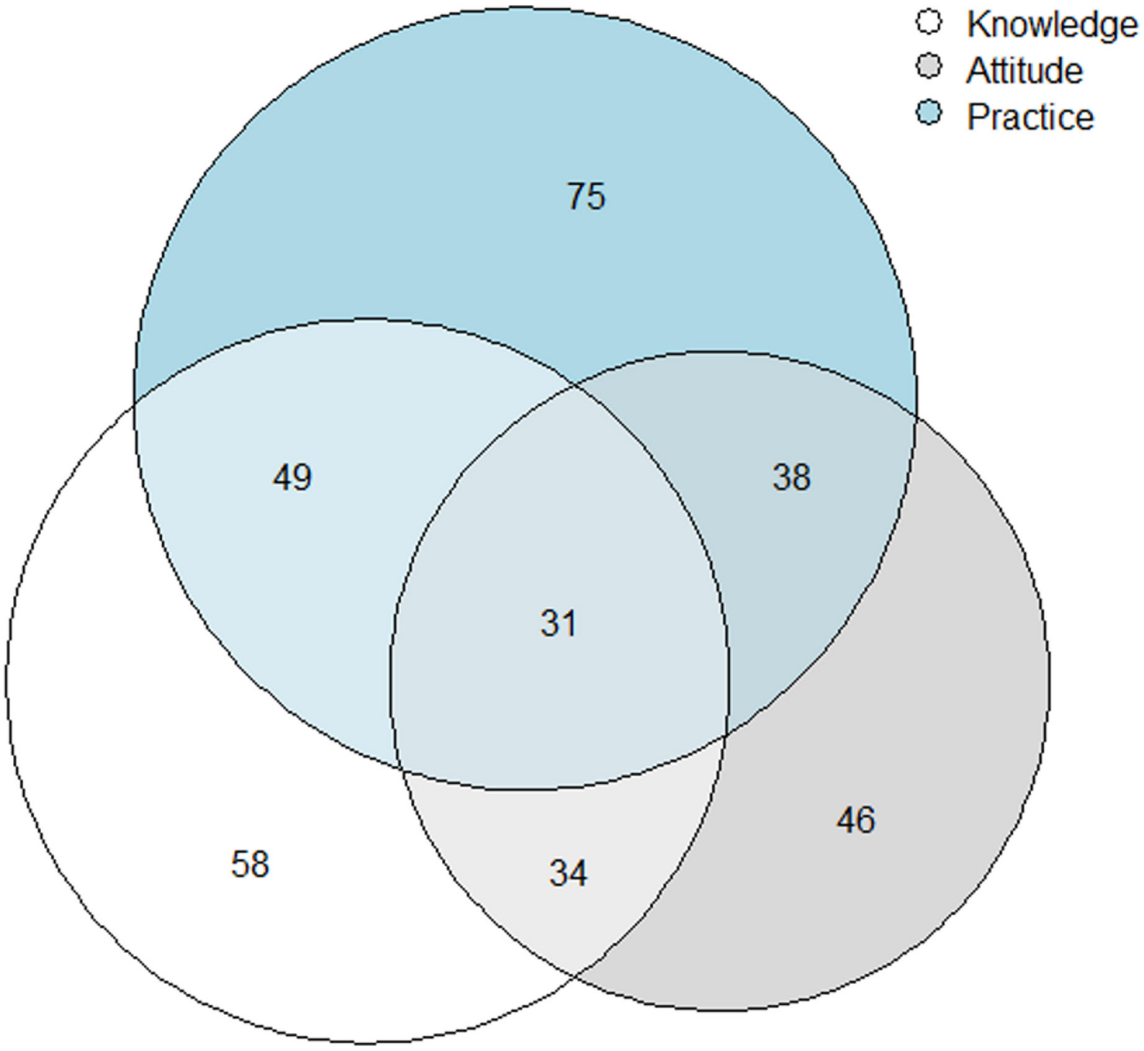
Venn diagram showing the knowledge, attitude, and practice of dengue prevention.

Figure 1 shows the Venn diagram showing the knowledge, attitude, and practice on dengue prevention among police personnel. It was found that knowledge of dengue fever was 58%, attitude of dengue fever was 46%, and practice of dengue fever prevention was 75%. About 34% of police personnel had knowledge and attitude on dengue; 49% of police personnel had knowledge and practice on dengue; 49% of police personnel had attitude and practice on dengue. About 31% of police personnel had knowledge, attitude, and practice on dengue prevention.

Table 2 shows the factors associated with knowledge on dengue among police personnel in both bivariate and multivariate analysis. Table 2 presents the number and percentage of participants with inadequate and adequate knowledge of dengue, as well as the *P*‐values, crude odds ratios (COR), and adjusted odds ratios (AOR) with their respective 95% confidence intervals (CI). In the bivariate analysis, several factors were significantly associated with knowledge of dengue, including gender, history of dengue among family members, good drainage system inside the household, the water storage containers were covered tight, and the availability of public water taps nearby. These factors were further analyzed in the multivariate analysis, and the adjusted odds ratios were presented. The results of the multivariate analysis showed that the history of dengue among family members, ownership of a bed net, and having covered water storage containers remained significantly associated with knowledge of dengue after being adjusted with other factors. The results show that females had higher odds of having knowledge on dengue fever compared to males, although this difference was not statistically significant after adjusting for other factors (adjusted odds ratio [AOR] = 1.89, 95% CI = 0.92–3.88). Having a history of dengue among family members was significantly associated with higher odds of having knowledge on dengue fever (AOR = 2.78, 95% CI = 1.38‐5.6). Ownership of a bed net and having covered water storage containers were significantly associated with higher odds of having knowledge on dengue fever (AOR = 2.13, 95% CI = 1.04‐4.35 and AOR = 2.99, 95% CI = 1.74‐5.13, respectively).

**TABLE 2:** Factors associated with knowledge on dengue among police personnel in bivariate and multivariate analyses.

Characteristics	Knowledge on dengue		*P*‐value	COR (95% CI)	AOR (95% CI)
	Inadequate	Adequate			
	Number (172.42%)	Number (239.58%)			
Gender			.012		
Male	160 (44.1)	203 (55.9)		Ref	Ref
Female	12 (25)	36 (75)		2.36 (1.19, 4.69)	1.89 (0.92, 3.88)
Age			.525		
Mean (*SD*)	31.4 (8.9)	32.1 (10.3)			
Religion			.271		
Hindu	163 (42.4)	221 (57.6)			
Buddhist	7 (43.8)	9 (56.2)			
Others	2 (18.2)	9 (81.8)			
Caste			.412		
Upper caste groups	29 (44.6)	36 (55.4)			
Janajatis	61 (38.4)	98 (61.6)			
Dalits	43 (40.6)	63 (59.4)			
Madhesi	16 (41)	23 (59)			
Others	23 (54.8)	19 (45.2)			
Number of family member			.065	Ref	Ref
Mean (*SD*)	6.1 (2.3)	5.7 (2.3)		0.92 (0.85, 1.01)	0.92 (0.84, 1.01)
Education			.244		
SEE/SLC and below	79 (45.1)	96 (54.9)			
Proficiency level certificate and above	93 (39.4)	143 (60.6)			
Ownership of bed net			.005		
No	20 (55.6)	16 (44.4)		Ref	Ref
Yes	152 (40.5)	223 (59.5)		1.83 (1.92, 3.65)	2.13 (1.04, 4.35)
History of dengue among family members			.026		
No	158 (43.9)	202 (56.1)		Ref	Ref
Yes	14 (27.5)	37 (72.5)		2.07 (1.08, 3.96)	2.78 (1.38, 5.6)
Good drainage system inside household			.001		
No	61 (55)	50 (45)		Ref	Ref
Yes	111 (37)	189 (63)		2.08 (1.34, 3.23)	1.16 (0.68, 1.98)
Natural light inside the house			.383		
No	22 (47.8)	24 (52.2)			
Yes	150 (41.1)	215 (58.9)			
The house has stored water in containers			.909		
No	107 (41.6)	150 (58.4)			
Yes	65 (42.2)	89 (57.8)			
The water storage containers were covered tight			<.001		
No	68 (61.8)	42 (38.2)			
Yes	104 (34.6)	197 (65.4)		3.07 (1.95, 4.82)	2.99 (1.74, 5.13)
Presence of flower vases in the house			.874		
No	57 (41.3)	81 (58.7)			
Yes	115 (42.1)	158 (57.9)			
Presence of discarded tires around the house			.067		
No	134 (39.8)	203 (60.2)		Ref	Ref
Yes	38 (51.4)	36 (48.6)		0.63 (0.38, 1.04)	0.96 (0.53, 1.72)
Broken glasses/discarded plastic bottles were present in the house			.677		
No	134 (42.4)	182 (57.6)			
Yes	38 (40)	57 (60)			
Regular water supply			.913		
No	16 (41)	23 (59)			
Yes	156 (41.9)	216 (58.1)			
Availability of public water tap nearby			.07		
No	94 (46.3)	109 (53.7)		Ref	Ref
Yes	78 (37.5)	130 (62.5)		1.44 (0.97, 2.13)	1.25 (0.82, 1.92)
Presence of garbage disposal nearby the house			.463		
No	104 (40.5)	153 (59.5)			
Yes	68 (44.2)	86 (55.8)			

Table 3 provides the results of bivariate and multivariate analyses of factors associated with attitude toward dengue among police personnel. In the multivariate analysis, after adjusting for other factors, history of dengue among family members, broken glasses or discarded plastic bottles were present in the house, and knowledge on dengue remained significantly associated with attitude toward dengue. Having a history of dengue among family members was significantly associated with higher odds of having good attitude on dengue fever (AOR = 2.45, 95% CI = 1.24‐4.87). Broken glasses or discarded plastic bottles were present in the house were significantly associated with higher odds of having attitude on dengue fever (AOR = 2.07, 95% CI = 1.93‐5.36). Knowledge on dengue was associated with a good attitude on dengue prevention, the police personnel who had knowledge on dengue had a higher attitude of dengue, with an AOR of 3.3 (95% CI: 2.09‐5.36).

**TABLE 3:** Factors associated with attitude on dengue among police personnel in bivariate and multivariate analyses.

Characteristics	Attitude on dengue		*P*‐value	COR (95% CI)	AOR (95% CI)
	Poor	Good			
	Number (224.54%)	Number (187.46%)			
Gender			.057		
Male	204 (56.2)	159 (43.8)		Ref	Ref
Female	20 (41.7)	28 (58.3)		1.8 (0.98, 3.31)	1.62 (0.82, 3.19)
Age			.801		
Mean (*SD*)	31.9 (9.1)	31.7 (10.4)			
Religion			.553		
Hindu	212 (55.2)	172 (44.8)			
Buddhist	7 (43.8)	9 (56.2)			
Others	5 (45.5)	6 (54.5)			
Caste			.141		
Upper caste groups	29 (44.6)	36 (55.4)			
Janajatis	83 (52.2)	76 (47.8)			
Dalits	59 (55.7)	47 (44.3)			
Madhesi	26 (66.7)	13 (33.3)			
Others	27 (64.3)	15 (35.7)			
Number of family member			.117		
Mean (*SD*)	6 (2.2)	5.7 (2.4)		0.93 (0.86, 1.02)	0.94 (0.86, 1.04)
Education			.783		
SEE/SLC and below	94 (53.7)	81 (46.3)			
Proficiency level certificate and above	130 (55.1)	106 (44.9)			
Ownership of bed net			.894		
No	20 (55.6)	16 (44.4)			
Yes	204 (54.4)	171 (45.6)			
History of dengue among family members			.001		
No	207 (57.5)	153 (42.5)		Ref	Ref
Yes	17 (33.3)	34 (66.7)		2.71 (1.46, 5.02)	2.45 (1.24, 4.87)
Good drainage system inside household			.58		
No	69 (62.2)	42 (37.8)			
Yes	155 (51.7)	145 (48.3)			
Natural light inside the house			.737		
No	24 (52.2)	22 (47.8)			
Yes	200 (54.8)	165 (45.2)			
The house has stored water in containers			.549		
No	143 (55.6)	114 (44.4)			
Yes	81 (52.6)	73 (47.4)			
The water storage containers were covered tight			.004		
No	73 (66.4)	37 (33.6)		Ref	Ref
Yes	151 (50.2)	150 (49.8)		1.96 (1.24, 3.09)	1.6 (0.9, 2.84)
Presence of flower vases in the house			.01		
No	63 (45.7)	75 (54.3)		Ref	Ref
Yes	161 (59)	112 (41)		0.58 (0.39, 0.88)	0.49 (0.31, 0.78)
Presence of discarded tires around the house			.667		
No	182 (54)	155 (46)			
Yes	42 (56.8)	32 (43.2)			
Broken glasses or discarded plastic bottles were present in the house			.039		
No	181 (57.3)	135 (42.7)		Ref	Ref
Yes	43 (45.3)	52 (54.7)		1.62 (1.02, 2.57)	2.07 (1.22, 3.52)
Regular water supply			.15		
No	17 (43.6)	22 (56.4)		Ref	Ref
Yes	207 (55.6)	165 (44.4)		0.62 (0.32, 1.2)	0.74 (0.35, 1.54)
Availability of public water tap nearby			.207		
No	117 (57.6)	86 (42.4)			
Yes	107 (51.4)	101 (48.6)			
Presence of garbage disposal nearby the house			.672		
No	138 (53.7)	119 (46.3)			
Yes	86 (55.8)	68 (44.2)			
Knowledge on dengue			<.001		
No	126 (73.3)	46 (26.7)		Ref	Ref
Yes	98 (41)	141 (59)		3.94 (2.58, 6.03)	3.3 (2.09, 5.19)

Table 4 provides the results of bivariate and multivariate analyses of factors associated with practice toward dengue among police personnel. In the multivariate analysis, after adjusting for other factors, water storage containers were covered tight, presence of flower vases in the house, knowledge on dengue, and attitude on dengue remained significantly associated with practice on dengue. The presence of flower vases in the house is associated with good practice on dengue prevention in the bivariate analysis, with an AOR of 1.68 (95% CI: 1.02, 2.77). The police personnel who had knowledge on dengue had higher odds of good practice on dengue prevention, with an AOR of 3.21 (95% CI: 1.93, 5.36). Attitude on dengue was also significantly associated with good practice on dengue prevention [AOR of 1.62 (95% CI: 0.96, 2.73)]; however, there was no association in the multivariate analysis.

**TABLE 4:** Factors associated with practice on dengue prevention among police personnel in bivariate and multivariate analyses.

Characteristics	Practice on dengue		*P*‐value	COR (95% CI)	AOR (95% CI)
	Poor	Good			
	Number (*N* = 101.25%)	Number (*N* = 310.75%)			
Gender			.319		
Male	92 (25.3)	271 (74.7)			
Female	9 (18.8)	39 (81.2)			
Age			.1		
Mean (*SD*)	33.2 (10.3)	31.3 (9.5)		0.98 (0.96, 1)	0.98 (0.96, 1)
Religion			.879		
Hindu	96 (25)	288 (75)			
Buddhist	3 (18.8)	13 (81.2)			
Others	2 (18.2)	9 (81.8)			
Caste			.734		
Upper caste groups	17 (26.2)	48 (73.8)			
Janajatis	38 (23.9)	121 (76.1)			
Dalits	29 (27.4)	77 (72.6)			
Madhesi	10 (25.6)	29 (74.4)			
Others	7 (16.7)	35 (83.3)			
Number of family member			.243		
Mean (*SD*)	6.1 (2.2)	5.8 (2.4)			
Education			.642		
SEE/SLC and Below	41 (23.4)	134 (76.6)			
Proficiency level certificate and above	60 (25.4)	176 (74.6)			
Ownership of bed net			.383		
No	11 (30.6)	25 (69.4)			
Yes	90 (24)	285 (76)			
History of dengue among family members			.853		
No	89 (24.7)	271 (75.3)			
Yes	12 (23.5)	39 (76.5)			
Good drainage system inside household			.742		
No	26 (23.4)	85 (76.6)			
Yes	75 (25)	225 (75)			
Natural light inside the house			.23		
No	8 (17.4)	38 (82.6)			
Yes	93 (25.5)	272 (74.5)			
The house has stored water in containers			.252		
No	68 (26.5)	189 (73.5)			
Yes	33 (21.4)	121 (78.6)			
The water storage containers were covered tight			.071		
No	34 (30.9)	76 (69.1)		Ref	Ref
Yes	67 (22.3)	234 (77.7)		1.56 (0.96, 2.54)	1.12 (0.66, 1.91)
Presence of flower vases in the house			.086		
No	41 (29.7)	97 (70.3)		Ref	Ref
Yes	60 (22)	213 (78)		1.5 (0.94, 2.39)	1.68 (0.82, 2.77)
Presence of discarded tires around the house			.401		
No	80 (23.7)	257 (76.3)			
Yes	21 (28.4)	53 (71.6)			
Broken glasses or discarded plastic bottles were present in the house			.238		
No	82 (25.9)	234 (74.1)			
Yes	19 (20)	76 (80)			
Regular water supply			.58		
No	11 (28.2)	28 (71.8)			
Yes	90 (24.2)	282 (75.8)			
Availability of public water tap nearby			.798		
No	51 (25.1)	152 (74.9)			
Yes	50 (24)	158 (76)			
Presence of garbage disposal nearby the house			.501		
No	66 (25.7)	191 (74.3)			
Yes	35 (22.7)	119 (77.3)			
Knowledge on dengue			<.001		
No	66 (38.4)	106 (61.6)		Ref	Ref
Yes	35 (14.6)	204 (85.4)		3.63 (2.26, 5.82)	3.21 (1.93, 5.36)
Attitude on dengue			.001		
No	69 (30.8)	155 (69.2)		Ref	Ref
Yes	32 (17.1)	155 (82.9)		2.16 (1.34, 3.47)	1.62 (0.96, 2.73)

## 4 DISCUSSION

To our knowledge, it is the first study among police personnel to determine knowledge, attitude, and practice toward dengue prevention and its associated factors among police personnel in Kathmandu valley. The study found that the knowledge of dengue fever was 58%, attitude of dengue fever was 46% and practice of dengue fever prevention was 75%. While dengue is a significant public health problem in many tropical and subtropical regions including Nepal, the level of knowledge and attitude toward dengue prevention is often low as suggested in similar studies of Nepal.^7^, ^11^, ^12^, ^13^ The reasons for inadequate knowledge on dengue fever can be due to various reasons, including cultural beliefs, lack of awareness, and ineffective communication strategies. Studies identified that people may not be aware of the signs and symptoms of dengue, its mode of transmission, and the importance of preventive measures, due to a lack of health education and ineffective communication strategies.^7^, ^13^, ^14^ However, the practice of dengue prevention was comparatively high due to the availability of preventive measures, the fear of contracting dengue, and government and public health interventions. The availability of preventive measures such as mosquito repellents, bed nets, and insecticides can encourage people to practice dengue prevention. When people have access to effective preventive measures, they are more likely to use them. As a result of dengue outbreaks in 2019 with high mortality,^5^, ^14^, ^15^ there has been a substantial increase in practice on dengue prevention and treatment options. The fear of contracting dengue motivates people to practice dengue prevention. When people are aware of the risks associated with dengue and the potential consequences of contracting the disease, they are more likely to take preventive measures. Government and public health interventions such as campaigns to raise awareness, the distribution of mosquito nets and repellents, and the implementation of mosquito control measures were on rise during the outbreaks.

The study also found that police personnel with a history of dengue among family members were more knowledgeable and attitude toward dengue fever than those without such a history.^4^, ^16^ This could be because those who have experienced the disease among themselves, and their families are more likely to be aware of the symptoms and preventive measures. They are likely to have a higher level of knowledge and attitude toward the disease due to their personal and family experience and the impact it has had on their lives.^10^, ^13^ They may develop a heightened awareness of the importance of preventing and controlling the disease and use proactive measures to reduce their risk of contracting the disease again, such as using mosquito repellents, wearing protective clothing, and eliminating mosquito breeding sites around their home. Having personal and family experience with dengue fever can also increase empathy and understanding toward others who have been affected by the disease.

Ownership of bed nets was associated with higher knowledge on dengue fever as consisted with similar studies.^17^, ^18^ The police personnel were aware that owning a bed net can help prevent dengue fever by reducing the exposure of individuals to the mosquitoes that transmit the virus. Bed nets are an effective tool for preventing mosquito bites, which can lead to the transmission of dengue.^19^

Knowledge and practice on environment factors associated with dengue was strong predictor with knowledge, attitude, and practice on dengue prevention.^8^, ^20^, ^21^ Having covered water storage containers were significantly associated with higher odds of having knowledge on dengue fever and broken glasses or discarded plastic bottles were present in the house were significantly associated with higher odds of having attitude on dengue fever. Understanding the environmental factors that contribute to dengue transmission is essential for effective prevention strategies.^20^ These factors include standing water, poor sanitation, and inadequate waste management, which provide breeding sites for mosquitoes. Knowledge of these factors enables individuals and communities to take steps to eliminate mosquito breeding sites and reduce the risk of dengue transmission.^8^, ^9^ Knowledge alone is not sufficient; individuals and communities must take action to prevent the spread of dengue.^22^ This includes measures such as removing standing water from their surroundings, using mosquito repellents, and wearing protective clothing. By practicing these behaviors, individuals and communities can reduce the risk of dengue transmission and protect themselves and others from infection.^8^, ^22^

The study found that police personnel who were more knowledgeable about dengue had a more positive attitude toward dengue.^7^, ^13^ This finding is consistent with the notion that knowledge is an important predictor of behavior change. Studies have shown that people who have a good knowledge of dengue are more likely to use mosquito repellents, wear protective clothing, and clean up mosquito breeding sites.^7^, ^10^, ^11^, ^12^, ^13^, ^14^ Furthermore, individuals who have a high level of knowledge about dengue are more likely to seek medical help early and reduce the risk of complications.^12^, ^13^

Attitudes toward dengue prevention are shaped by factors such as beliefs, social norms, and cultural practices.^23^ People who have a good understanding of dengue and have positive attitudes toward prevention are more likely to take action to prevent the disease.^10^, ^11^, ^16^ In contrast, individuals who lack knowledge or have negative attitudes toward dengue prevention are less likely to take preventive measures, which can lead to an increased risk of dengue transmission.

The association between knowledge, attitude, and practice is essential for effective dengue prevention and control.^13^, ^16^ Studies have shown that individuals who have a good level of knowledge about dengue are more likely to have positive attitudes toward prevention and take effective preventive measures.^14^, ^15^, ^16^, ^17^, ^18^, ^19^, ^20^ Similarly, people with positive attitudes toward dengue prevention are more likely to take preventive measures, even if they do not have a high level of knowledge about the disease.^17^, ^18^, ^19^ Effective preventive practices require the integration of knowledge and positive attitudes toward dengue prevention.^9^, ^20^

Several studies have examined the relationship between knowledge, attitude, and practice and dengue prevention and control. For example, studies conducted in Nepal^7^, ^12^, ^13^ and India^18^, ^19^, ^20^ found that people who had a high level of knowledge about dengue were more likely to use mosquito repellent and clean up mosquito breeding sites. People who had positive attitudes toward dengue prevention were more likely to take preventive measures, such as using mosquito repellent and cleaning up mosquito breeding sites.^17^, ^18^, ^23^ These findings highlight the importance of knowledge, attitude, and practice of dengue prevention in dengue prevention and control.

There are some limitations to this study that should be taken into account when interpreting the results. Firstly, the study relied on self‐report measures, which are subject to response bias and social desirability bias. Participants may have been reluctant to report certain behaviors or thoughts due to social norms or stigma. Secondly, the study was cross‐sectional, meaning that it captured a snapshot of participants' experiences at one point in time and not possible to establish causal relationships between variables or to determine the direction of the observed relationships.

## 5 CONCLUSION

In conclusion, the study found that the level of knowledge and attitude toward dengue prevention among police personnel in Kathmandu valley was low, but the practice of dengue prevention was comparatively moderate. The availability of preventive measures, fear of contracting dengue, and government and public health interventions contributed to this. Personal and family experience with dengue fever, ownership of bed nets, and knowledge of environmental factors associated with dengue were also associated with higher levels of knowledge, attitude, and practice toward dengue prevention. Knowledge, attitude, and practice are all essential for effective dengue prevention and control, and their integration is necessary. Effective preventive practices require individuals and communities to take action to prevent the spread of dengue, including measures such as removing standing water, using mosquito repellents, and wearing protective clothing. It is recommended that health education campaigns be conducted to increase awareness and knowledge about dengue fever. The campaigns should be designed in a culturally appropriate manner and should utilize effective communication strategies. Therefore, it is recommended that efforts be made to improve environmental hygiene and sanitation, and to educate the public on the importance of eliminating mosquito breeding sites.

## 6 AUTHOR CONTRIBUTIONS

SK, DP and NL contributed to the concept and design, analysis and interpretation of data, drafted and revised the manuscript. SK and JD involve in data analysis and interpretation. DP involved in acquisition of data and SR, DC and RPS involved in drafting the manuscript and revising it critically for intellectual content. All the authors read and approved the final manuscript.

## DISCLOSURE

The authors declare that there is no conflict of interest.

## Data Availability

Research data are not shared.
